# Langerhan Cell Histiocytosis: A Rare Disorder With a Rare Presentation

**DOI:** 10.14740/wjon880w

**Published:** 2015-04-12

**Authors:** Divya Byragani, Krishna Baradhi, Igor Schendrick, Supriya Koya

**Affiliations:** a University of Oklahoma School of Community Medicine, Tulsa, OK 74135, USA; b Division of Nephrology and Hypertension, University of Oklahoma School of Community Medicine, Tulsa, OK 74135, USA; c University of Oklahoma, Regional Medical Laboratory, 4142 South Mingo Rd., Tulsa, OK 74146, USA; d Utica Park Clinic Oncology, 1245 S Utica Ave Ste 240, Tulsa, OK 74104, USA

**Keywords:** Langerhans cell histiocytosis, Rare presentation

## Abstract

Langerhans cell histiocytosis (LCH) is a rare disorder most commonly seen in Caucasians of Northern European decent, male, children. The most common presentation is osteolytic bone lesions. A 44-year-old native American presents with diffuse erythematous, scaling lesions. The patient also had pruritus and lymphadenopathy. These lesions were positive for S-100 and CD1a. The patient was started on chemotherapy which improved her symptoms immensely. This was a rare disease with a rare presentation.

## Introduction

Langerhans cell histiocytosis (LCH) is a rare histocytic disorder mostly prevalent in the neonatal age, with a peak incidence between 1 and 3 years of age. There is a higher incidence in males, which becomes less obvious with increasing age and most common in whites of northern European descent. LCH commonly presents with osteolytic bone lesions and less commonly in liver, spleen, lymph nodes and bone marrow [1]. We present a 44-year-old native American woman with erythema, scaling, hyperpigmentation of her palms and soles associated with intense pruritus and lymphadenopathy. Immunohistochemical panel demonstrated a histiocytic population with uniformly positive staining with S-100 and CD1a, suggesting LCH.

## Case Report

A 44-year-old native American female with a history of diabetes, hypertension, hypothyroidism, depression and hyperlipidemia presented to the Indian clinic after she noticed increased itching with associated maculopapular rash on her flank as well as upper and lower extremities.

**Figure 1:**
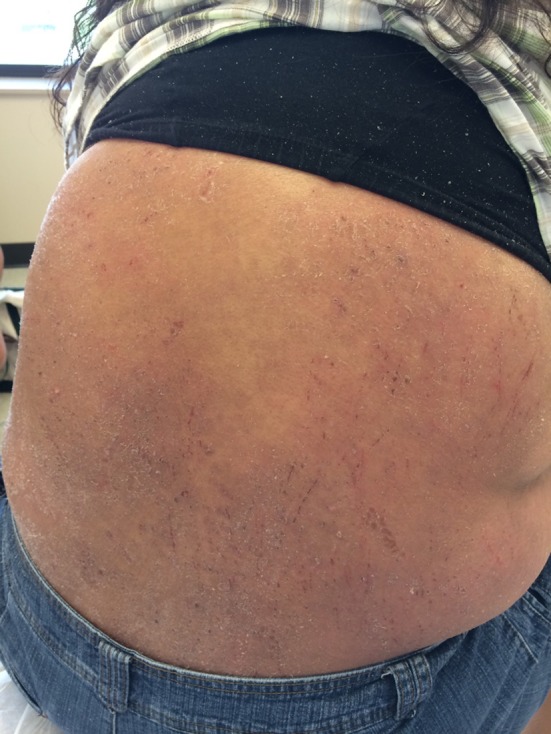
Excoriation and lesions on patient’s flank on presentation.

**Figure 2:**
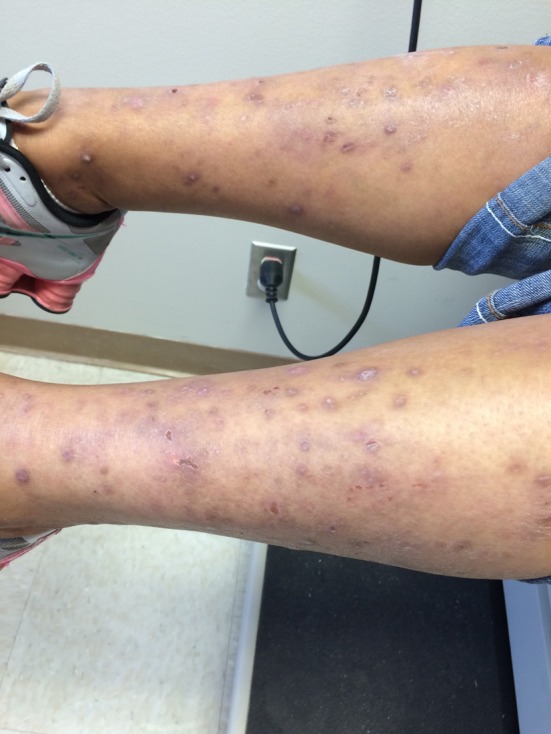
Excoriations and lesions on legs before treatment

Initially, the skin rash was attributed to a streptococcus infection and was treated with appropriate antibiotics. However, she returned back to clinic with high fevers and worsening rash and was found to have a leukocytosis of 30,000/µL prompting admission to the hospital and treatment with intravenous antibiotics (Fig. 1, 2). Patient was discharged after 2 weeks with some resolution of her symptoms.

Patient was readmitted 1 month later with persistent leukocytosis and worsening rash. At this time, examination showed erythema of her palms and soles as well as erythema, scaling and hyperpigmentation scattered all over her extremities and trunk with associated axillary and inguinal lymphadenopathy. Labs showed WBC of 23,000/µL with eosinophilia of 8,000/µL. A CT scan of the chest, abdomen and pelvis showed an 11 mm right axillary lymph node, 12 mm pre-tracheal lymph node, 12 mm pre-carinal lymph node, as well as multiple enlarged retroperitoneal lymph nodes. A needle biopsy of the axillary lymph node was inconclusive prompting axillary lymph node excisional biopsy. Bone scan is negative for any bone lesions.

**Figure 3:**
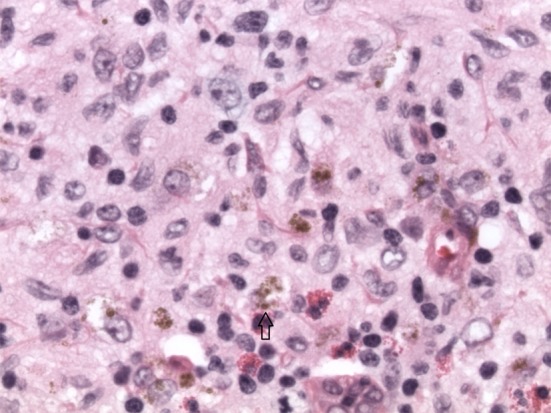
H&E image × 40 magnification. Langerhans cells with scattered eosinophils. Arrow points to a Langerhans cell.

**Figure 4:**
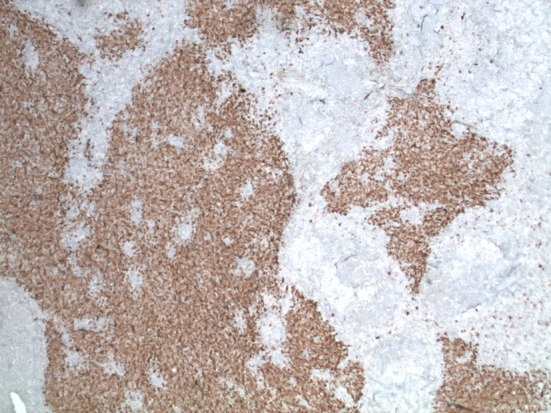
Immunohistochemical stain with S-100, magnification × 2. The Langerhans cells are S-100 positive.

Biopsy showed enlarged lymph node, demonstrating expanded and irregular germinal centers, surrounded by small lymphocytes. Large clusters of histiocytes intermixed with numerous eosinophils and focally containing pigment, suggestive of dermatopathic lymphadenopathy component, expanded the sinuses. Some of the histiocytic cells demonstrate presence of groups and prominent nucleolus, raising the differential diagnosis of LCH. Further evaluation with immunohistochemical stains revealed histiocytic population, which was strongly and uniformly positive for S-100 and CD1a, supporting the diagnosis of LCH (Fig. 3, 4). The lesion also demonstrated significant number of CD30 positive cells. Further immunohistochemical staining with CD15, CD 45, CD20, and CD3 was performed to exclude Hodgkin’s lymphoma and demonstrate the atypical CD30 positive cells to be positive with Langerhan cell antigen (CD45) and focally positive with CD20.

**Figure 5:**
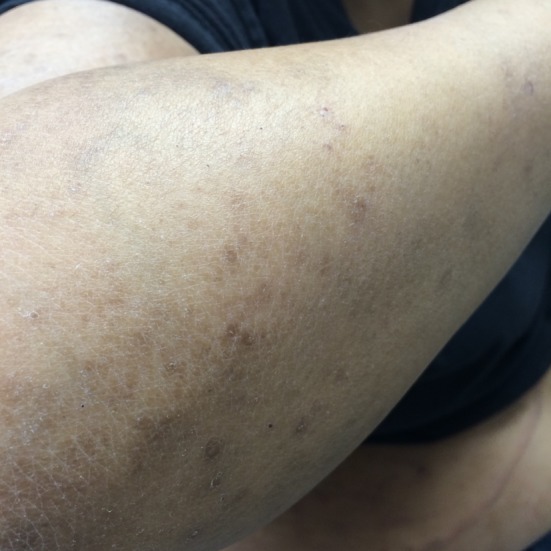
Lesions after therapy.

**Figure 6:**
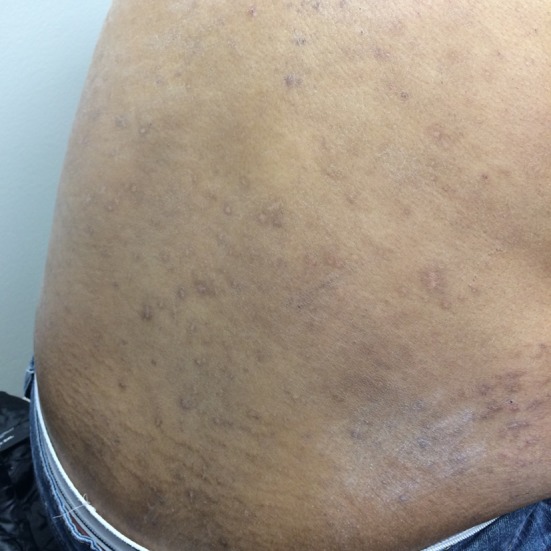
Lesions after therapy.

What was originally thought to be a drug-induced reaction was confirmed by lymph node biopsy to be LCH. This is an uncommon presentation of a rare disease. The diagnosis of LCH was discussed with the patient and was started on chemotherapy initially with vinblastine and later switched to etoposide and prednisone with continued improvement in her rash and systemic symptoms (Fig. 5, 6).

## Discussion

LCH is a clonal proliferative disorder of histocytes that pathologically accumulate in various organs. Langherhan cells are usually present in the skin, spleen and lymph nodes. As these cells resemble dendritic antigen-presenting Langerhans cells, it is called LCH. These antigen presenting cells can be characterized into a spectrum of histiocytic disorder which involves a combination of mononuclear-phagocyte system and Langerhans cell proliferation [2, 3]. Though it is thought that LCH is a benign condition, it can be argued that clonal proliferation suggests a malignant process [3].

LCH is most commonly seen in northeastern European male children and presents as bone lesions. Several retrospective studies have shown that 51-71% of children with LCH present with multiorgan disease; immunohistochemical stains show these cells are positive for S-100, Fc receptor, CD1 [4, 5]. Our patient was found to have stains positive for S100, CD1a and CD30. The etiology of LCH remains unknown; research has shown links between Epstein-Barr virus, malaria and leukemia [5].

The most common presentation in adults is skin lesions; it is known that LCH morphology differs from the Langerhans cells seen in the epidermis. These cells are large ovoid cells with coffee-bean nuclei and found in the epidermis and upper dermis [5]. These lesions can present as sebhorreic lesions, papules, purpura and xanthomas. Most commonly these lesions are found on the trunk and scalp and are rarely pruritic [6, 7]. Our patient presented with pruritic, erythematic, scaling hyperpigmented lesions associated with lymphadenopathy. Lymphadenopathy is a rare presentation of this disease and seen only in 4% of patients [6]. Risk stratification is critical as prognosis of LCH depends on involvement of multiple organ, and also response to chemotherapy during the initial months of therapy.

### Conclusion

LCH is characterized by multitude of clinical manifestations and biopsy is often required for definitive diagnosis. Atypical cases of LCH present diagnostic challenges and require a high index of suspicion. This case highlights the unusual presentation of a rare disease and teaches us an important lesson to consider diagnostic possibility of LCH as right diagnosis dictates correct treatment.
